# Multiparametric cardiovascular magnetic resonance is associated with outcomes in pediatric heart transplant recipients

**DOI:** 10.1016/j.jocmr.2024.101138

**Published:** 2024-12-25

**Authors:** Andrew A. Lawson, Kae Watanabe, Lindsay Griffin, Christina Laternser, Michael Markl, Cynthia K. Rigsby, Joshua D. Robinson, Nazia Husain

**Affiliations:** a Division of Cardiology, Department of Pediatrics, Ann & Robert H. Lurie Children’s Hospital of Chicago, Northwestern University Feinberg School of Medicine, Chicago, Illinois, USA; b Division of Cardiology, Department of Pediatrics, Baylor College of Medicine, Houston, Texas, USA; c Department of Radiology, Connecticut Children’s Medical Center, Hartford, Connecticut, USA; d Center for Cardiovascular Innovation, Ann & Robert H. Lurie Children’s Hospital of Chicago, Chicago, Illinois, USA; e Department of Radiology, Northwestern University Feinberg School of Medicine, Chicago, Illinois, USA; f Department of Medical Imaging, Ann & Robert H. Lurie Children’s Hospital of Chicago, Chicago, Illinois, USA

**Keywords:** Pediatric heart transplant, T1 mapping, T2 mapping, Extracellular volume fraction, Late gadolinium enhancement, Graft surveillance

## Abstract

**Background:**

Multiparametric cardiovascular magnetic resonance (CMR) has an emerging role in non-invasive surveillance of pediatric heart transplant recipients (PHTR). Higher myocardial T2, higher extracellular volume fraction (ECV), and late gadolinium enhancement (LGE) have been associated with adverse clinical outcomes in adult heart transplant recipients. The purpose of this study was to investigate the prognostic value of CMR-derived T1 and T2 mapping, ECV, and LGE for clinical outcomes in PHTR.

**Methods:**

We performed a single-center, retrospective chart review of consecutive, gadolinium-enhanced CMR studies in PHTR over a 7.5-year period, excluding follow-up studies. Standard CMR ventricular volume and function analysis, T1 mapping with ECV, T2 mapping, and LGE assessment were performed. The composite outcome included cardiac death, non-cardiac death, re-transplantation, and cardiac hospitalization.

**Results:**

Among 113 PHTR, mean age was 13.0 ± 5.1 years, with 6.0 ± 4.0 years since transplant. The indication for CMR was surveillance in 79%. Mean native T1 was 1050 ± 48 ms, T2 49.2 ± 3.9 ms, and ECV 29.7 ± 4.5%. Left ventricular LGE was present in 37% (42/113) and right ventricular LGE in 3.5% (4/113). The mean follow-up time was 2.3 years and median was 1.4 years. Cardiac death occurred in 2% (2/113), re-transplantation in 4% (4/113), and cardiac hospitalization in 22% (25/113). Non-cardiac death did not occur. Using Kaplan-Meier analysis, high T1 (≥1061 ms), T2 (≥50.0 ms), and ECV (≥31.4%) were each associated with decreased freedom from the composite outcome in follow-up. In univariable Cox regression analyses, high T1 was associated with increased risk of the composite outcome (hazard ratios [HR] 4.0, 95% confidence interval [CI] 1.7–9.2, p = 0.001), as were high T2 (HR 2.8, 95% CI 1.1–7.1, p = 0.026), and high ECV (HR 3.5, 95% CI 1.5–8.1, p = 0.004).

**Conclusion:**

T1 and T2 mapping are associated with early differences in adverse cardiac events in PHTR. These data suggest a role for a multicenter study with a longer follow-up duration.

## Graphical abstract

**Figure:**
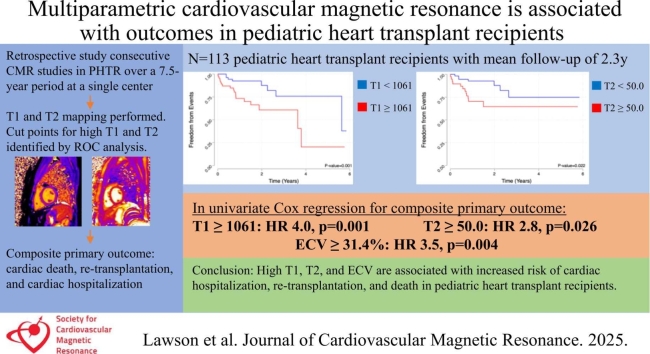

## 1 Background

Survival after pediatric heart transplantation has continued to improve with advances in post-transplant care despite increases in the comorbidities of patients selected for transplantation [1], [2]. The overall median survival after pediatric heart transplantation is over 18 years [3], which highlights the considerable progress in the field as well as the stark difference in expected survival between pediatric heart transplant recipients (PHTR) and healthy children. The leading causes of death after pediatric heart transplantation differ based on the time since transplantation, but graft failure is the leading cause of death at all time points [3].

The role of cardiovascular magnetic resonance (CMR) in the surveillance of heart transplant recipients is an area of active research. CMR offers precise ventricular volumetric and function data, as well as myocardial tissue characterization with quantification of myocardial T1 and T2, extracellular volume fraction (ECV), and assessment of late gadolinium enhancement (LGE). Studies of adult heart transplant recipients undergoing concurrent endomyocardial biopsy and CMR have demonstrated increased native T1 [4], [5], [6], T2 [5], [7], [8], [9], and ECV [4], [7] in patients with acute rejection by histopathological criteria. These studies reported generally high negative predictive values of normal mapping parameters for acute rejection. This evidence suggests a role for CMR in ruling out acute rejection non-invasively. A comparatively smaller number of studies in PHTR have similarly found that biopsy-proven rejection is associated with higher T1 [10], [11], T2 [11], [12], [13], and ECV [11]. The role of T1 and T2 mapping for cardiac allograft vasculopathy (CAV) has been studied less, though the available data have demonstrated that heart transplant recipients with CAV have increased T1 [14], T2 [15], [16], and ECV [14] compared to those without CAV.

Collectively, these studies demonstrate that CMR detects myocardial tissue changes reflective of significant graft pathology in heart transplant recipients. Longitudinal data are still required to establish the prognostic ability of CMR mapping. In adult heart transplant recipients, high T2 [17], ECV [17], and LGE [18], [19], [20], [21] have been associated with decreased freedom from subsequent cardiac events. These associations with longitudinal outcomes have not been studied in PHTR. The purpose of this study was to evaluate the prognostic value of CMR-based tissue characterization with T1 and T2 mapping, ECV, and LGE for adverse cardiac events in a large single-center cohort of PHTR.

## 2 Methods

### 2.1 Study design and patient selection

This is a retrospective, longitudinal cohort study of PHTR who underwent CMR at a single center from 2015–2022. The Institutional Review Board (IRB) approved this study. All PHTR with a clinician-ordered CMR during the study period were included. The first CMR observation during the study period was included and follow-up studies were excluded. All patients were <18 years of age at the time of transplant and received care at a pediatric center, but patients were not excluded based on age at the time of CMR.

### 2.2 CMR protocol

CMR studies were performed on 1.5T Siemens scanners (Aera, Siemens Healthineers, Erlangen, Germany) according to a standardized institutional protocol with comprehensive structure and function assessment. Gadobutrol (Gadavist; Bayer HealthCare; Berlin, Germany) was used as the gadolinium-based contrast agent. T1 mapping was performed using a modified Look-Locker inversion recovery 3,3,5 sequence with adjustment for heart rate at three short-axis slices (base, mid, and apex) pre- and post-contrast with single short balanced steady-state free precession (bSSFP) readout. T2 mapping was performed using three T2-prepped bSSFP images with varying T2-prep times (0, 24, and 55 ms) in the same three short-axis slice positions. LGE was obtained using segmented inversion-recovery sequences in three orientations: four-chamber, two-chamber, and short axis from base to apex 20–30 min from the time of the initial contrast injection. Inversion time was selected using a scout scan to optimally null the normal myocardium. Please refer to the previously described protocol for details of image acquisition [14]. During the study period, no changes were made to the T1 or T2 sequences. All exams were performed on the same scanner. Institutional protocol changed during the study period to include T2 mapping: this change accounts for some missing T2 data from patients who underwent CMR early in the study period.

### 2.3 CMR postprocessing

Volumetric and function analysis: Cine SSFP data were used for CMR-derived volume and function analysis including right ventricular and left ventricular end-diastolic volume indexed to body surface area (RVEDVI and LVEDVI), end-systolic volumes indexed to body surface area (RVESVI and LVESVI), ejection fractions (RVEF and LVEF), and left ventricular (LV) mass indexed to body surface area (LVMI) using commercially-available software (Q Mass, Medis Suite 4.0.38.2, Medis Medical Imaging Systems, Leiden, The Netherlands).

#### 2.3.1 T1 mapping, ECV, and T2 mapping

Epicardial and endocardial contours were manually traced on each motion-corrected T2 map, native T1 map, and post-contrast T1 map, excluding the blood pool and pericardial fat. A region of interest was placed in the center of the blood pool on pre- and post-contrast T1 images. Native T1, T2, and post-contrast T1 values were calculated for each segment according to the American Heart Association 16-segment model, as recommended by the Society for Cardiovascular Magnetic Resonance guidelines [22]. ECV was calculated for each segment using the standard formula [23]. Global native T1, T2, and ECV represent the average of all segmental values. T1 and T2 mapping were not performed in rare cases when images were of insufficient quality for analysis. Two reviewers (L.G. and N.H.) assessed the presence and location of qualitative LV and RV LGE in consensus by visual identification of areas of relatively increased signal intensity on delayed, contrast-enhanced images. Orthogonal planes were used to confirm the presence of LGE. Native T1, T2, and ECV values were compared to previously reported institutional normative data from 39 healthy children without a history of heart transplantation (T1 989.6 ± 34.3 ms, ECV 23.3 ± 2.4%, T2 46.7 ± 2.6 ms) [24].

### 2.4 Clinical data collection

By chart review, a single reviewer (A.L.) collected baseline and follow-up outcomes data. Baseline patient data (events before CMR) included patient demographics, indication for transplantation, donor and graft characteristics, rejection history, cardiac catheterization data, and history of CAV. The indication for CMR was classified as either surveillance or a clinical concern. Since 2020, our center has performed CMR in surveillance of low-risk patients without significant rejection history or CAV, which allows these patients to skip one annual cardiac catheterization with the plan to perform a cardiac catheterization the next year. In other cases, CMR was performed for clinical concern (e.g. history of rejection, CAV, changes on echocardiogram, or changes in catheterization-derived hemodynamic parameters). Patients with clinical suspicion of rejection still underwent cardiac catheterization and biopsy and were not treated on the basis of CMR results unless multiple vessel occlusions prohibited vascular access for biopsy. During chart review, we reviewed both the CMR order and the clinical notes written by the heart transplant team before CMR to determine if there was a clinical concern. Patients were classified as undergoing CMR either without sedation or with general anesthesia. History of moderate or severe rejection was defined as any episode of biopsy-proven acute cellular rejection (ACR) ≥2R or antibody-mediated rejection (AMR) ≥2, per International Society of Heart and Lung Transplantation (ISHLT) criteria before CMR [25], [26]. If a hemodynamic catheterization and endomyocardial biopsy were performed within 6 months of CMR, invasive hemodynamic data and biopsy results were included as baseline data; otherwise, data were reported missing. The cardiac index was calculated by the estimated Fick method with LaFarge estimates of oxygen consumption [27]. A history of CAV was defined as any angiographic CAV (grade 1–3) by ISHLT criteria as assessed by the interventional cardiologist performing the procedure, of cardiac catheterization before or within 12 months following the CMR [28].

The composite outcome includes cardiac death, non-cardiac death, re-transplantation, and cardiac hospitalization (such as admission for treatment of clinical rejection, arrhythmia, or evaluation of cardiac symptoms). Outcome data were collected by chart review. Events were included if they occurred after CMR and after the clinical encounter or hospitalization in which CMR occurred.

### 2.5 Statistical analysis

Descriptive statistics are presented as mean ± SD for normally distributed variables. To test for significance, we used a t-test for continuous variables and chi-square test for categorical variables (Stata 17, College Station, Texas). Given the absence of institutional normative data for myocardial mapping parameters in pediatric transplant recipients, we elected to identify cut points for high T1, T2, and ECV in this population using separate, nonparametric analyses of receiver operating characteristic (ROC) curves for native T1, T2, and ECV. Cut points were then selected using the Liu function to maximize the product of the sensitivity and specificity of each parameter from the composite outcome. Using these cut points, we performed Kaplan-Meier analyses to evaluate differences in freedom from the composite outcome as a function of time based on high or low native T1, T2, or ECV, and presence of LGE. Patients reached the composite clinical outcome through the first qualifying event. Univariate Cox regression analyses were used to evaluate differences in time to the composite clinical outcome based on CMR-derived mapping, volume, and function parameters. Multivariate Cox regression analyses were performed to evaluate differences in the composite clinical outcome based on T1, T2, ECV, and LGE in a combined model as well as in separate models with one mapping parameter adjusted for other clinically relevant baseline CMR parameters.

## 3 Results

### 3.1 Patient clinical and CMR characteristics

The cohort included 113 PHTR (51% female), with a mean age at CMR of 13.0 ± 5.1 years, with 6.0 ± 4.0 years since transplant. The indication for CMR was routine surveillance in 79% (89/113) of studies; a clinical concern prompted the CMR in remaining 21% (24/113). For studies prompted by a clinical concern, the most commonly stated indications were history of rejection (n = 13), hemodynamic catheterization findings (n = 4), echocardiogram findings (n = 3), and history of CAV (n = 3). In 26% of studies (29/113), CMR was performed with general anesthesia. The cohort had relatively low rate of graft complications, with 25% (28/113) having history of moderate or severe rejection and 7% (8/113) with history of CAV. The mean baseline hemodynamic parameters were normal, with cardiac index of 4.0 ± 1.0 L/min/m^2^, right atrial pressure 6.0 ± 3.4 mmHg, and pulmonary capillary wedge pressure 11.1 ± 4.9 mmHg. Baseline clinical characteristics are presented in Table 1.

**Table 1:** Baseline patient characteristics and CMR findings for all patients, and for the subsets of patients who reached or did not reach the composite outcome in follow-up.

	N*	All patients	Composite outcome: yes	Composite outcome: no	p-value
*Patient and graft characteristics*					
Age, y, mean ± SD	113	13.0 ± 5.1	13.7 ± 4.3	12.8 ± 5.3	0.446
Female sex, n (%)	113	58 (51)	16 (64)	42 (47)	0.151
Time since transplant, y, mean ± SD	113	6.0 ± 4.0	5.2 ± 3.2	6.2 ±0 .2	0.289
Donor age at transplant, y, mean±SD	101	8.5 ± 7.5	11.0 ± 7.8	7.7 ± 7.4	0.064
*Indication for transplant*					
Cardiomyopathy, n (%)	113	43 (38)	8 (32)	35 (40)	0.698
Congenital heart disease, n (%)		64 (57)	16 (64)	48 (55)	
Re-transplantation, n (%)		6 (5)	1 (4)	5 (6)	
*Transplant comorbidities*					
History of moderate or severe AMR or ACR, n (%)	112	28 (25)	13 (52)	15 (17)	**<0.001**
History of any CAV, n (%)	113	8 (7)	7 (28)	1 (1)	**<0.001**
Clinical concern present, n (%)	113	24 (21)	12 (48)	12 (14)	**<0.001**
Cardiac catheterization data (if performed within 6 months of CMR)					
Cardiac index, L/min/m^2^, mean ± SD	84	4.0 ± 1.0	3.7 ± 1.1	4.1 ± 1.1	0.175
Right atrial pressure, mmHg, mean ± SD	83	6.0 ± 3.4	7.0 ± 4.2	5.7 ± 3.1	0.133
Pulmonary capillary wedge pressure, mmHg, mean ± SD	84	11.1 ± 4.9	12.0 ± 6.2	10.9 ± 4.3	0.344
AMR on biopsy, n (%)	84	5 (6)	2 (11)	3 (5)	0.297
ACR 1R, n (%)	84	16 (19)	4 (22)	12 (18)	0.699
ACR ≥ 2R, n (%)	84	1 (1)	0 (0)	1 (2)	0.599

*AMR* antibody-mediated rejection, *ACR* acute cellular rejection, *CAV* cardiac allograft vasculopathy, *CMR* cardiovascular magnetic resonance, *SD* standard deviation

Data are presented as mean ± SD or as n (%), as indicated in the relevant table row. Bold p-values denote statistical significance.

* Total cohort N = 113 patients. Variation in total N is based on available data.

The observed mean RV and LV volumes and ejection fractions were normal. Global native T1, T2, and ECV were 1049.8 ± 48.3 ms, 49.2 ± 3.9 ms, and 29.7 ± 4.5%, respectively. LV LGE was present in 42 (37%) and RV LGE in 4 (3.5%). Native T1 mapping was performed in 99% of CMR studies(112/113), ECV calculation in 98% (111/113), and T2 mapping in 91% (103/113). A summary of CMR findings is presented in Table 2.

**Table 2:** CMR findings.

	N*	Observed value
RVEDVI, mL/m^2^, mean ± SD	110	72.8 ± 15.9
RVEF, %, mean ± SD	110	55.5 ± 7.4
LVEDVI, mL/m^2^, mean ± SD	112	72.3 ± 14.5
LVEF, %, mean ± SD	112	59.1 ± 6.3
LVMI, g/m^2^, mean ± SD	108	46.2 ± 8.2
Global native T1, ms, mean ± SD	112	1049.8 ± 48.3
Global T2, ms, mean ± SD	103	49.2 ± 3.9
Global ECV, %, mean± SD	111	29.7 ± 4.5
Qualitative ventricular LGE present, n (%)	113	42 (37)
Qualitative LV LGE present, n (%)	113	42 (37)
Qualitative RV LGE present, n (%)	113	4 (3.5)

*RVEDVI* right ventricular end-diastolic volume index, *RVEF* right ventricular ejection fraction, *LVEDVI* left ventricular end-diastolic volume index, *LVEF* left ventricular ejection fraction, *LVMI* left ventricular mass index, *ECV* extracellular volume fraction, *LV* left ventricular, *LGE* late gadolinium enhancement, *RV* right ventricular, *SD* standard deviation, *CMR* cardiovascular magnetic resonance

Data are presented as mean ± SD or as n (%), as indicated in the relevant table row.

* Total cohort N = 113 patients. Variation in total N is based on available data

### 3.2 Clinical outcomes

The mean follow-up time was 2.3 ± 1.9 years, median 1.4 years (interquartile range 0.9–3.0 years). The composite outcome occurred in 22% (25/113). Cardiac death occurred in 2% (2/113), re-transplantation in 4% (4/113), and cardiac hospitalization in 22% of patients (25/113). Non-cardiac death did not occur. All patients who died or were re-transplanted reached the composite outcome endpoint through cardiac hospitalizations occurring before these events. History of CAV, history of moderate or severe rejection, or a clinical concern prompting the CMR study were each associated with increased risk of reaching the composite outcome during the follow-up period (Table 1).

### 3.3 Associations of CMR findings with future clinical events

**Fig. 1:**
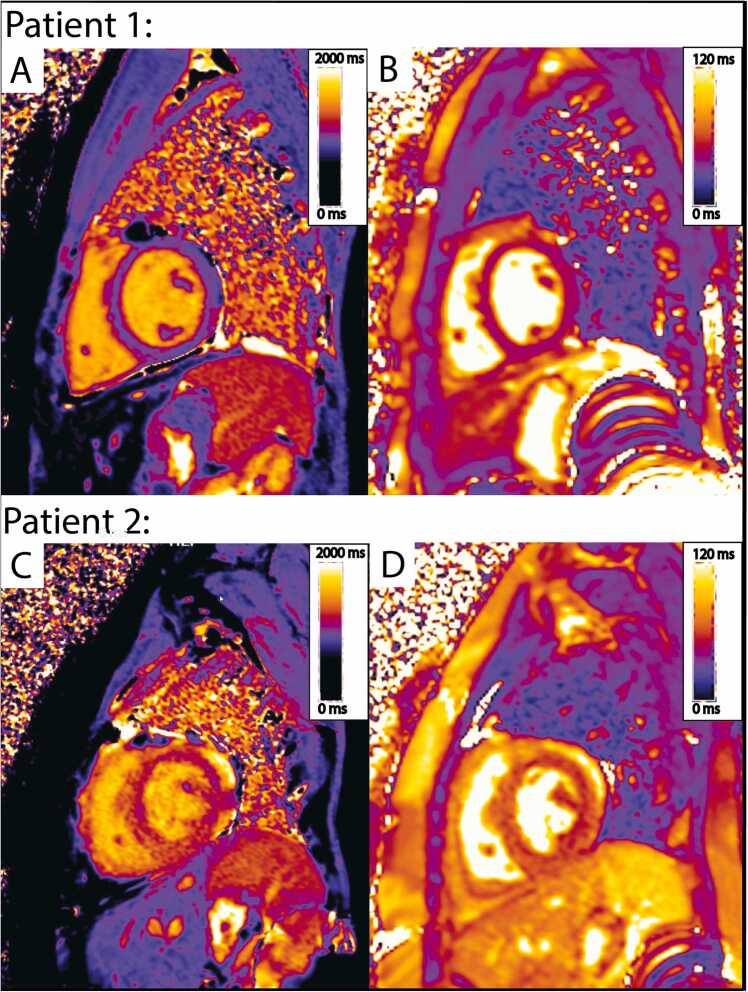
Patient 1 is a 14-year-old male, 3-year post-transplant, with CMR performed for routine surveillance in lieu of annual catheterization. CMR demonstrated normal myocardial T1 of 985 ms (A) and T2 of 47 ms (B). No adverse clinical events were observed in follow-up. Patient 2 is a 19-year-old female, 10-year post-transplant, who underwent CMR for a clinical concern (palpitations and elevated donor-specific antibody). CMR demonstrated abnormal mapping, with global native T1 of 1175 ms (C) and T2 of 60 ms (D). Seven months after CMR, the patient suffered a cardiac arrest. She subsequently received a second heart transplant. *CMR* cardiovascular magnetic resonance

ROC analysis identified the optimal cut points for native T1, T2, and ECV for the composite outcome as T1 ≥1061 ms, T2 ≥50.0 ms, and ECV ≥31.4%, with areas under the curve of 0.64, 0.70, and 0.55, respectively. Examples of patients from the study cohort with normal and abnormal T1 and T2 mapping are provided in Fig. 1.

By univariate Cox regression analyses, high T1 (≥1061 ms) was associated with increased risk of reaching the composite outcome (hazard ratios [HR] 4.0, 95% confidence interval [CI] 1.7–9.2, p = 0.001), as were high T2 (≥50.0 ms) (HR 2.8, 95% CI 1.1–7.1, p = 0.026), and high ECV (≥31.4%) (HR 3.5, 95% CI 1.5–8.1, p = 0.004). The presence of LGE was not associated with a statistically significant difference in the composite outcome (HR 1.6, 95% CI 0.8–3.6, p = 0.213). RV and LV end-diastolic volume indices, ejection fractions, and LVMI were not associated with a statistically significant difference in outcomes. The univariate Cox regression analyses are given in Table 3.

**Table 3:** Univariate Cox regression analysis for the composite outcome.

	Hazard ratio (95% CI)	p-value
RVEDVI, mL/m^2^	1.002 (0.976–1.029)	0.879
RVEF, %	1.001 (0.956–1.049)	0.940
LVEDVI, mL/m^2^	1.020 (0.991–1.049)	0.177
LVEF, %	0.973 (0.897–1.056)	0.518
LVMI, g/m^2^	1.026 (0.967–1.089)	0.394
Global native T1 ≥1061 ms	3.99 (1.73–9.20)	**0.001**
Global T2 ≥50.0 ms	2.83 (1.13–7.09)	**0.026**
Global ECV ≥31.4%	3.48 (1.50–8.08)	**0.004**
LGE+	1.63 (0.75–3.57)	0.213

*RVEDVI* right ventricular end-diastolic volume index, *RVEF* right ventricular ejection fraction, *LVEDVI* left ventricular end-diastolic volume index, *LVEF* left ventricular ejection fraction, *LVMI* left ventricular mass index, *ECV* extracellular volume fraction, *LGE* late gadolinium enhancement, *CI* confidence interval

Hazard ratios are followed by 95% confidence intervals in parentheses. Bold p-values denote statistical significance.

In a multivariate regression analysis including T1, ECV, T2, and LGE, only T2 retained a significant positive association with the composite outcome (β = 0.045, p = 0.001). In separate multivariate regression analyses—each containing one mapping parameter (T1, T2, or ECV) and adjusted for LVEF and LVMI—we found that high native T1, high T2, and high ECV remained associated with increased risk of the composite outcome, independent of the LVEF and LVMI observed on CMR. Although LVEF and LVMI were not associated with the composite outcome in univariate analysis, these covariates were included in the multivariate analysis to ensure that mapping parameters were evaluated independently of graft structural and function parameters at the time of CMR. These results are provided in Table 4.

**Table 4:** Multivariate Cox regression analyses for the composite outcome, with mapping parameters adjusted for LVEF and LVMI*.

	Hazard ratio (95% CI)	p-value
Global native T1 ≥1061 ms	4.37 (1.78–10.73)	**0.001**
Global T2 ≥50.0 ms	3.46 (1.35–8.85)	**0.010**
Global ECV ≥31.4%	3.60 (1.50–8.60)	**0.004**
LGE+	1.58 (0.73–3.43)	0.246

*LVEF* left ventricular ejection fraction, *LVMI* left ventricular mass index, *ECV* extracellular volume fraction, *LGE* late gadolinium enhancement, *CI* confidence interval

Hazard ratios are followed by 95% confidence intervals in parentheses. Bold p-values denote statistical significance.

* Each row represents a separate multivariate regression including the specified mapping parameter, LVEF, and LVMI

**Fig. 2:**
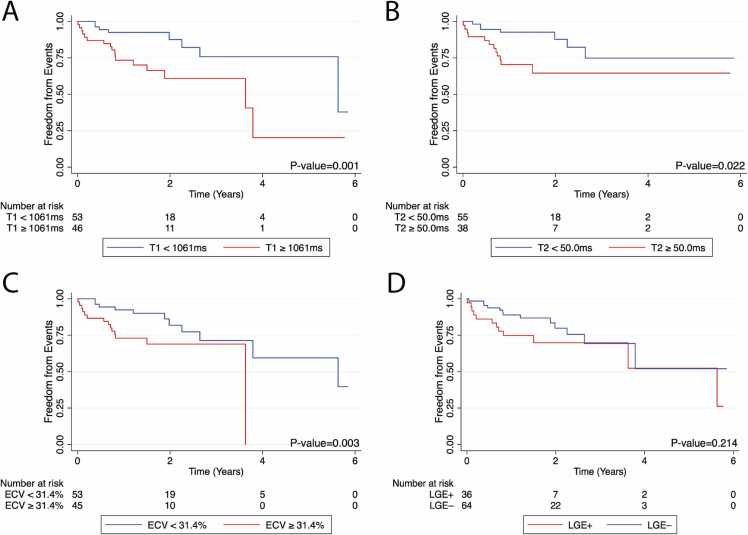
Kaplan-Meier survival curves for pediatric heart transplant recipients with (A) high global native T1 (≥1061 ms), (B) high global T2 (≥50.0 ms), (C) high global ECV (≥31.4%), and (D) presence of LGE, for the composite outcome of cardiac death, non-cardiac death, re-transplantation, and cardiac hospitalization. *ECV* extracellular volume fraction, *LGE* late gadolinium enhancement

By Kaplan-Meier analysis, high T1 (≥1061 ms), T2 (≥50.0 ms), and ECV (≥31.4%) were each associated with decreased freedom from the composite outcome, with p = 0.001, p = 0.022, and p = 0.003, respectively. There was no difference in freedom from the composite outcome on the basis of LGE (p = 0.214). Kaplan-Meier curves are provided in Fig. 2.

## 4 Discussion

This study demonstrates that PHTR with high T1, T2, and ECV are at increased risk of cardiac hospitalization, re-transplantation, and death. These associations are independent of graft function by LVEF at the time of CMR.

Chronic graft failure, acute rejection, and CAV are the leading causes of death in PHTR greater than 3 years post-transplant [3]. Previous work has established a role for T1 and T2 mapping for detecting myocardial tissue changes associated with acute rejection in PHTR [10], [11], [12], [13]. Limited work has suggested that CMR-derived mapping differences are also present in PHTR with CAV [14], [16]. Given these established associations between tissue changes as assessed by CMR and known causes of morbidity in transplant patients, the longitudinal outcomes demonstrated in this study are not surprising. Even so, establishing the prognostic ability of CMR for clinical outcomes is a significant and requisite step as the role of CMR in post-transplant surveillance continues to grow. We note that the observed outcomes occurred over a relatively short follow-up time (mean 2.3 years), which suggests that abnormal mapping findings have implications for risk of adverse outcomes even in the short term. The findings of this study support the continued use and study of CMR as a non-invasive means of surveillance in PHTR. We are hopeful that as our understanding of CMR mapping in PHTR advances, patients will ultimately require fewer cardiac catheterizations with endomyocardial biopsy and will instead undergo a regimen of non-invasive surveillance regimen including CMR and additional laboratory diagnostics, such as donor-derived cell-free DNA. [29], [30], [31].

The lack of standardization in absolute T1 and T2 values is a persistent challenge impeding both mapping research and the translation of this research to clinical practice. Absolute parametric values vary based on mode of acquisition, magnetic field strength, type of contrast used, and postprocessing technique [32]. The Society for Cardiovascular Magnetic Resonance has recommended the use of local, institution-specific reference ranges that were acquired, processed, and analyzed in the same way as the intended application [23]. Because the mean T1, T2, and ECV values in PHTR are different than those found in normal, healthy children [14], [33], [34], we elected to determine the cut points for elevated T1, T2, and ECV through an ROC analysis rather than use a predetermined threshold derived from normative pediatric control data from our institution. We note that the selected thresholds for each of these parameters were just slightly greater than the corresponding mean values in this cohort of PHTR and significantly greater than our previously published institutional normative values for healthy children. Given the many sources of variability in T1 and T2 values, investigators at other institutions will need to independently identify clinically relevant thresholds above which PHTR may be at higher risk. ECV may have better external validity due to its use of a ratio of blood and myocardial T1 signal.

The associations between high T2 and ECV and adverse cardiac events in this study corroborate the findings of a prior study on adult heart transplant recipients [17]. The association we observed between high native T1 and clinical outcomes has not been previously demonstrated in adult or PHTR. In multivariate analysis with all mapping parameters and LGE included, T2 remained associated with increased risk of the composite clinical outcome. We suspect T1 and ECV lost statistical significance in the multivariate analysis due to significant covariance between parameters and the low clinical event rate.

While previous adult studies have demonstrated that adult transplant recipients with LGE are at increased risk of future adverse events [18], [19], [20], [21], our study did not. LGE was fairly common in our cohort, though we observed variable patterns, previously described [35], and suspect certain patterns may be more clinically significant than others. A larger investigation with a longer follow-up duration is indicated to further study the clinical implications of LGE, including its variable patterns and extent, in PHTR. Future study should also investigate the significance and prognostic value of peak T1 and T2 values in addition to mean values. While establishing the prognostic significance of individual CMR findings in PHTR is essential, the study of the combined utility of multiple available non-invasive testing parameters, such as CMR and donor-derived cell-free DNA, will ultimately be most useful to clinicians and effective in reducing endomyocardial biopsy and improving outcomes in PHTR.

## 5 Limitations

The retrospective study design with inclusion of clinically ordered CMR studies creates selection bias. Not all PHTR at our center underwent CMR. The study included CMR studies ordered for surveillance and for clinical concerns, more often the former, which means that the cohort as a whole was relatively healthy, without high prevalence of pre-existing graft pathology such as acute rejection or CAV. Despite this, we found the significance of mapping in predicting graft and clinical outcomes. Multivariable Cox regression analyses were underpowered, limited by the number of clinical events observed and significant covariance between mapping parameters. As previously discussed, the lack of standardization of absolute mapping parameters limits the external validity of the reported T1 and T2 cut points above which we noted increased risk of clinical events.

## 6 Conclusions

High global myocardial T1, T2, and ECV are associated with an increased risk of adverse clinical events in the short-term follow-up of PHTR. These results suggest a role for a larger, multicenter study with longer follow-up duration to further investigate prognostic ability of CMR in surveillance of PHTR.

## Funding

Research reported in this publication was supported by the National Heart, Lung, And Blood Institute of the 10.13039/100000002National Institutes of Health under Award Number R01HL117888.

## Author contributions

A.A.L. and N.H. formulated the study and wrote the manuscript. A.A.L. collected the clinical data. N.H. and L.G. performed CMR image review. C.L. analyzed the data. All authors analyzed the results and reviewed the manuscript. All authors approved of the final manuscript.

## Ethics approval and consent

The study was approved by the Lurie Children’s Hospital and Northwestern University Feinberg School of Medicine IRB. The study was Health Insurance Portability and Accountability Act compliant and informed consent was waived.

## Consent for publication

Not applicable.

## Declaration of competing interests

Michael Markl reports financial support was provided by National Heart Lung and Blood Institute. Michael Markl reports a relationship with Siemens Healthineers AG that includes funding grants. Michael Markl reports a relationship with Circle Cardiovascular Imaging Inc. that includes funding grants. The other authors declare that they have no known competing financial interests or personal relationships that could have appeared to influence the work reported in this paper.

## Data Availability

The datasets used and/or analyzed during the current study are available from the corresponding author upon reasonable request.

## References

[1] Singh T.P., Cherikh W.S., Hsich E., Chambers D.C., Harhay M.O., Hayes D. (2021). The international thoracic organ transplant registry of the international society for heart and lung transplantation: twenty-fourth pediatric heart transplantation report - 2021; focus on recipient characteristics. J Heart Lung Transplant.

[2] Singh T.P., Cherikh W.S., Hsich E., Harhay M.O., Hayes D., Perch M. (2022). The international thoracic organ transplant registry of the international society for heart and lung transplantation: Twenty-fifth pediatric heart transplantation report-2022; focus on infant heart transplantation. J Heart Lung Transplant.

[3] Rossano J.W., Singh T.P., Cherikh W.S., Chambers D.C., Harhay M.O., Hayes D. (2019). The international thoracic organ transplant registry of the international society for heart and lung transplantation: twenty-second pediatric heart transplantation report - 2019; Focus theme: Donor and recipient size match. J Heart Lung Transplant.

[4] Sade L.E., Hazirolan T., Kozan H., Ozdemir H., Hayran M., Eroglu S. (2019). T1 mapping by cardiac magnetic resonance and multidimensional speckle-tracking strain byechocardiography for the Detection of Acute Cellular Rejection in Cardiac Allograft Recipients. JACC Cardiovasc Imaging.

[5] Anthony C., Imran M., Pouliopoulos J., Emmanuel S., Iliff J., Liu Z. (2022). Cardiovascular Magnetic Resonance for Rejection Surveillance After Cardiac Transplantation. Circulation.

[6] Imran M., Wang L., McCrohon J., Yu C., Holloway C., Otton J. (2019). Native T1 Mapping in the Diagnosis of Cardiac Allograft Rejection: A Prospective Histologically Validated Study. JACC Cardiovasc Imaging.

[7] Vermes E., Pantaleon C., Auvet A., Cazeneuve N., Machet M.C., Delhommais A. (2018). Cardiovascular magnetic resonance in heart transplant patients: diagnostic value of quantitative tissue markers: T2 mapping and extracellular volume fraction, for acute rejection diagnosis. J Cardiovasc Magn Reson.

[8] Usman A.A., Taimen K., Wasielewski M., McDonald J., Shah S., Giri S. (2012). Cardiac magnetic resonance T2 mapping in the monitoring and follow-up of acute cardiac transplant rejection: a pilot study. Circ Cardiovasc Imaging.

[9] Butler C.R., Savu A., Bakal J.A., Toma M., Thompson R., Chow K. (2015). Correlation of cardiovascular magnetic resonance imaging findings and endomyocardial biopsy results in patients undergoing screening for heart transplant rejection. J Heart Lung Transplant.

[10] Richmann D.P., Gurijala N., Mandell J.G., Doshi A., Hamman K., Rossi C. (2022). Native T1 mapping detects both acute clinical rejection and graft dysfunction in pediatric heart transplant patients. J Cardiovasc Magn Reson.

[11] Kikano S., Lee S., Dodd D., Godown J., Bearl D., Chrisant M. (2024). Cardiac magnetic resonance assessment of acute rejection and cardiac allograft vasculopathy in pediatric heart transplant. J Heart Lung Transplant.

[12] Soslow J.H., Godown J., Bearl D.W., Crum K., Slaughter J.C., George-Durrett K. (2022). Cardiac Magnetic Resonance Imaging Noninvasively Detects Rejection in Pediatric Heart Transplant Recipients. Circ Cardiovasc Imaging.

[13] Sethi N., Doshi A., Doshi T., Cross R., Cronin I., Amin E. (2020). Quantitative cardiac magnetic resonance T2 imaging offers ability to non-invasively predict acute allograft rejection in children. Cardiol Young.

[14] Husain N., Watanabe K., Berhane H., Gupta A., Markl M., Rigsby C.K. (2021). Multi-parametric cardiovascular magnetic resonance with regadenoson stress perfusion is safe following pediatric heart transplantation and identifies history of rejection and cardiac allograft vasculopathy. J Cardiovasc Magn Reson.

[15] Abbasi M.A., Blake A.M., Sarnari R., Lee D., Anderson A.S., Ghafourian K. (2022). Multiparametric Cardiac Magnetic Resonance Imaging Detects Altered Myocardial Tissue and Function in Heart Transplantation Recipients Monitored for Cardiac Allograft Vasculopathy. J Cardiovasc Imaging.

[16] Watanabe K., Arva N.C., Robinson J.D., Rigsby C., Markl M., Sojka M. (2024). Cardiac magnetic resonance imaging in detection of progressive graft dysfunction in pediatric heart transplantation. Pediatr Transplant.

[17] Chaikriangkrai K., Abbasi M.A., Sarnari R., Dolan R., Lee D., Anderson A.S. (2020). Prognostic Value of Myocardial Extracellular Volume Fraction and T2-mapping in Heart Transplant Patients. JACC Cardiovasc Imaging.

[18] Butler C.R., Kumar A., Toma M., Thompson R., Chow K., Isaac D. (2013). Late gadolinium enhancement in cardiac transplant patients is associated with adverse ventricular functional parameters and clinical outcomes. Can J Cardiol.

[19] Chaikriangkrai K., Abbasi M.A., Sarnari R., Lee D., Anderson A.S., Ghafourian K. (2019). Natural History of Myocardial Late Gadolinium Enhancement Predicts Adverse Clinical Events in Heart Transplant Recipients. JACC Cardiovasc Imaging.

[20] Hughes A, Okasha O, Farzaneh-Far A, Kazmirczak F, Nijjar PS, Velangi P (2019). Myocardial Fibrosis and Prognosis in Heart Transplant Recipients. Circ Cardiovasc Imaging.

[21] Pedrotti P., Vittori C., Facchetti R., Pedretti S., Dellegrottaglie S., Milazzo A. (2017). Prognostic impact of late gadolinium enhancement in the risk stratification of heart transplant patients. Eur Heart J Cardiovasc Imaging.

[22] Messroghli D.R., Moon J.C., Ferreira V.M., Grosse-Wortmann L., He T., Kellman P. (2017). Clinical recommendations for cardiovascular magnetic resonance mapping of T1, T2, T2* and extracellular volume: A consensus statement by the Society for Cardiovascular Magnetic Resonance (SCMR) endorsed by the European Association for Cardiovascular Imaging (EACVI). J Cardiovasc Magn Reson..

[23] Schulz-Menger J., Bluemke D.A., Bremerich J., Flamm S.D., Fogel M.A., Friedrich M.G. (2020). Standardized image interpretation and post-processing in cardiovascular magnetic resonance - 2020 update : Society for Cardiovascular Magnetic Resonance (SCMR): Board of Trustees Task Force on Standardized Post-Processing. J Cardiovasc Magn Reson.

[24] Cornicelli M.D., Rigsby C.K., Rychlik K., Pahl E., Robinson J.D. (2019). Diagnostic performance of cardiovascular magnetic resonance native T1 and T2 mapping in pediatric patients with acute myocarditis. J Cardiovasc Magn Reson.

[25] Berry G.J., Burke M.M., Andersen C., Bruneval P., Fedrigo M., Fishbein M.C. (2013). The 2013 International Society for Heart and Lung Transplantation Working Formulation for the standardization of nomenclature in the pathologic diagnosis of antibody-mediated rejection in heart transplantation. J Heart Lung Transplant.

[26] Stewart S., Winters G.L., Fishbein M.C., Tazelaar H.D., Kobashigawa J., Abrams J. (2005). Revision of the 1990 working formulation for the standardization of nomenclature in the diagnosis of heart rejection. J Heart Lung Transplant.

[27] LaFarge CG, Miettinen OS (1970). The estimation of oxygen consumption. Cardiovascular Research.

[28] Mehra M.R., Crespo-Leiro M.G., Dipchand A., Ensminger S.M., Hiemann N.E., Kobashigawa J.A. (2010). International Society for Heart and Lung Transplantation working formulation of a standardized nomenclature for cardiac allograft vasculopathy-2010. J Heart Lung Transplant.

[29] Khush K.K., Patel J., Pinney S., Kao A., Alharethi R., DePasquale E. (2019). Noninvasive detection of graft injury after heart transplant using donor-derived cell-free DNA: A prospective multicenter study. Am J Transplant.

[30] Agbor-Enoh S., Shah P., Tunc I., Hsu S., Russell S., Feller E. (2021). Cell-Free DNA to Detect Heart Allograft Acute Rejection. Circulation.

[31] Feingold B., Rose-Felker K., West S.C., Miller S.A., Zinn M.D. (2023). Short-term clinical outcomes and predicted cost savings of dd-cfDNA-led surveillance after pediatric heart transplantation. Clin Transplant.

[32] Rao S., Tseng S.Y., Pednekar A., Siddiqui S., Kocaoglu M., Fares M. (2022). Myocardial Parametric Mapping by Cardiac Magnetic Resonance Imaging in Pediatric Cardiology and Congenital Heart Disease. Circ Cardiovasc Imaging.

[33] Ide S., Riesenkampff E., Chiasson D.A., Dipchand A.I., Kantor P.F., Chaturvedi R.R. (2017). Histological validation of cardiovascular magnetic resonance T1 mapping markers of myocardial fibrosis in paediatric heart transplant recipients. J Cardiovasc Magn Reson.

[34] Riesenkampff E., Chen C.K., Kantor P.F., Greenway S., Chaturvedi R.R., Yoo S.J. (2015). Diffuse Myocardial Fibrosis in Children After Heart Transplantations: A Magnetic Resonance T1 Mapping Study. Transplantation.

[35] Lawson A.A., Watanabe K., Griffin L., Laternser C., Markl M., Rigsby C.K. (2023). Late-gadolinium enhancement is common in older pediatric heart transplant recipients and is associated with lower ejection fraction. J Cardiovasc Magn Reson.

